# Study of Spectrally Resolved Thermoluminescence in Tsarev and Chelyabinsk Chondrites with a Versatile High-Sensitive Setup

**DOI:** 10.3390/ma14216518

**Published:** 2021-10-29

**Authors:** Alexander Vokhmintsev, Ahmed Henaish, Taher Sharshar, Osama Hemeda, Ilya Weinstein

**Affiliations:** 1 NANOTECH Center, Ural Federal University, 620002 Ekaterinburg, Russia; a.s.vokhmintsev@urfu.ru (A.V.); a.Henaish@urfu.ru (A.H.); 2 Physics Department, Faculty of Science, Tanta University, Tanta 31527, Egypt; omhemeda@science.tanta.edu.eg; 3 Physics Department, Faculty of Science, Kafrelshaikh University, Kafr El-Shaikh 33735, Egypt; taher.sharshar@hotmail.com; 4 Institute of Metallurgy of the Ural Branch of the Russian Academy of Sciences, 620016 Ekaterinburg, Russia

**Keywords:** TL spectroscopy, ordinary chondrite, Chelyabinsk LL5, Tsarev L5, activation energy

## Abstract

Thermoluminescence (TL) research provides a powerful tool for characterizing radiation-induced processes in extraterrestrial matter. One of the challenges in studying the spectral features of the natural TL of stony meteorites is its weak intensity. The present work showcases the capabilities of a high-sensitive original module for measuring the spectrally resolved TL characteristics of the Chelyabinsk and Tsarev chondrites. We have analyzed the emission spectra and glow curves of natural and induced TL over the 300–650 nm and RT–873 K ranges. A quasi-continuous distribution of traps active within the 350–650 K range was found in the silicate substructure of both meteorites under study. Based on the general order kinetic formalism and using the natural TL data, we also estimated the activation energies of E_A_ = 0.86 and 1.08 eV for the Chelyabinsk and Tsarev chondrites, respectively.

## 1. Introduction

Thermoluminescence (TL) spectroscopy is a well-proven experimental method for studying the spectral characteristics and kinetic mechanisms of radiation-stimulated processes in irradiated materials [1,2]. In practice, there are different TL-based techniques to apply in archaeological and geological dating for dose exposure and radiation contamination monitoring by commercial systems, and different techniques correspond to specific targets within solid-state dosimetry using luminescent technologies [3]. Moreover, TL research provides a large-yield stomping ground for characterizing thermal history and describes various impact events, metamorphic processes, and features of the inorganic composition in extraterrestrial materials [4]. Catching the low intensity of natural TL is an arduous enough issue in exploring the spectral properties of meteorites. Usually, an integral luminescent response in a wide wavelength range is detected only. However, this method of studying meteorites that exhibit simultaneous emission in different bands is not always effective [5,6]. Therefore, one does not succeed in analyzing the spectral peculiarities of the natural and induced TL at a required spectral resolution. Previously, we designed and put in practice a high-temperature module as a supplementary unit for commercial fluorescence spectrometers. Additionally, its performance was tested within the range of up to 773 K for temperature, using wide-gap nitrides as examples [7,8]. In this paper, we demonstrate the capabilities of an original high-temperature module by measuring the spectrally resolved thermoluminescence of the Chelyabinsk and Tsarev meteorites. The goal is to evaluate spectral and energy parameters of thermally stimulated processes in the chondrites exposed by the high-dose irradiation.

## 2. Materials and Methods

**Figure 1:**
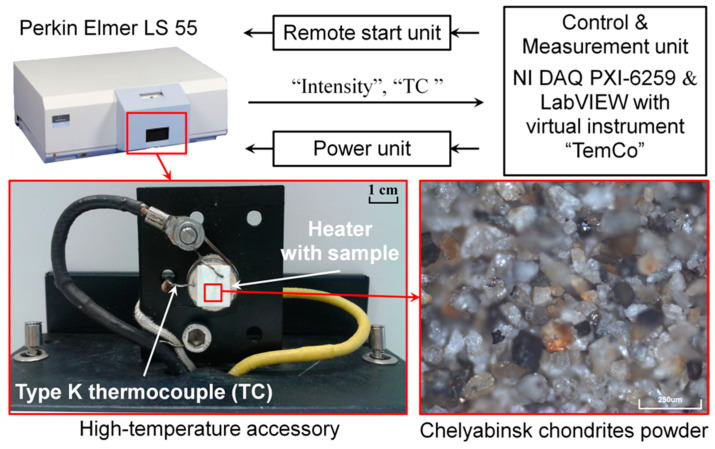
Thermoluminescent spectrometer.

Several fragments of the Chelyabinsk LL5 (fall date is 15 February 2013) and Tsarev L5 (fall date is 6 December 1922) chondrites have been studied. The meteorite cores were refined from the fusion crust, followed by grinding into a micro-sized powder. Later on, we applied hydrochloric acid treatment to the latter to remove metal particles (see Figure 1).

The thermoluminescence measurements of the samples were carried out in the phosphorescence regime (12.5 ms of gate time and 20 ms of cycle time) using a LS 55 Perkin Elmer spectrometer with an original heating accessory module [7]. Figure 1 presents a block diagram of the developed TL spectrometer. The latter includes four main parts: a high-temperature accessory with a heating stage and a thermocouple, a power unit, a remote start unit, and a control and measurement unit. A detailed configuration and the operating regimes of the experimental setup, as well as its abilities to study thermally activated luminescence processes in wide-gap materials, were described in References [7,8].

The natural and induced TL glow curves for both meteorites were recorded for the 440 ± 20 nm band within the RT–873 K range with a linear heating rate of r = 2 K/s. An UELR-10-15S linear accelerator with 10 MeV electrons was utilized for the irradiation of the samples and excited an induced TL response. The radiation doses amounted to 9.1–36.4 kGy. For subsequent numerical processing, 4 measurements of the TL glow curves were performed for each value of the dose.

The TL spectra ranged from 300 to 650 nm and RT–873 K were analyzed with a scanning speed of 700 nm/min and r = 0.5 K/s. About 15 spectra were recorded during the single heating process, while the temperature of the sample changed by 15 K within one measurement of the spectral dependence. In this case, a starting temperature of the recording was assigned to each spectrum. Figures with TL spectra and TL glow curves show the selection of the measured dependencies, accounting for the clarity and completeness of the experimental data. The spectral parameters of natural TL for the Tsarev meteorite could not be analyzed due to its very low emission intensity.

## 3. Results and Discussion

**Figure 2:**
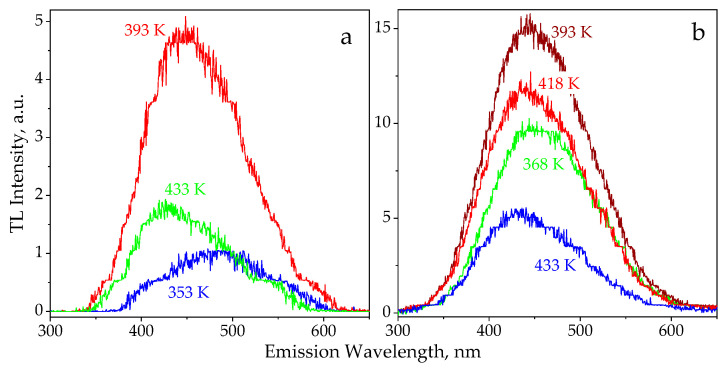
Emission spectra of natural TL in the Chelyabinsk chondrite (**a**) and induced TL in the Tsarev chondrite (**b**).

**Figure 3:**
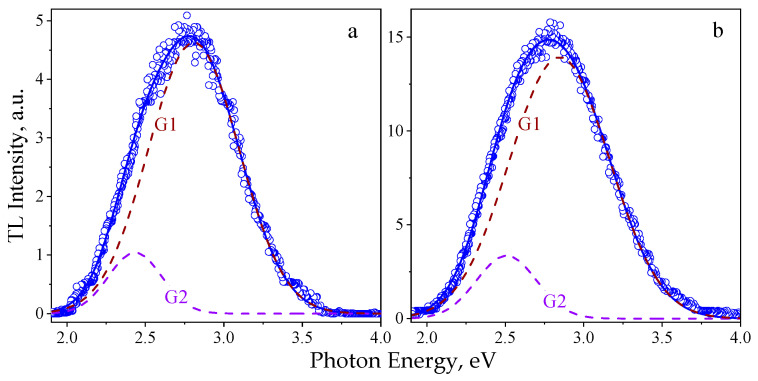
Deconvolution of TL spectra measured at 393 K for the Chelyabinsk (**a**) and Tsarev (**b**) chondrites. For both plots: circles—experimental data; colored dashed lines—the corresponding Gaussian components; and solid blue lines—resulting approximation curves.

Figure 2 shows experimental spectra of the natural and induced TL for the Chelyasbinsk and Tsarev chondrites, respectively. A wide structureless band in the visible spectral range was observed in the TL emission for both meteorites under investigation at the indicated temperatures. All the TL spectra could be approximated with high accuracy (coefficient of determination is R^2^ > 0.993) by a superposition of two G_1_ and G_2_ Gaussians; see Figure 3. For the appropriate temperatures, the G1 dominated in the TL emission, as its intensity was four to six times higher than that of the G_2_ peak. The values of the E_max_ maximum energies and ω_E_ half-widths for the Gaussian bands are presented in Table 1 in comparison with independent data on spectral parameters of photo (PL) and cathodoluminescence (CL) for the same chondrites.

Previous studies of the Chelyabinsk [6] and Tsarev [9] meteorites have shown that the observed PL emission spectra are also characterized by two Gaussians. The shape of the PL bands did not change, while their intensity decreased with varying excitation photon energy within the 6.2–4.5 eV range. The values of the spectral parameters obtained for the G_1_ and G_2_ components suggest that the PL and TL processes are due to the same recombination centers in both meteorites. In turn, the CL spectrum for the Chelyabinsk meteorite contained only a single Gaussian-shaped band and its parameters were consistent with the G_1_ component; see Table 1. It can be concluded that the TL, CL, and PL spectra have the same emission composition, which indicates the similarity of the recombination centers involved in the mechanisms of the luminescence of the investigated meteorites exposed by UV, electrons, and space irradiation.

Analysis of the data obtained and independent studies allows one to conclude that the observed features of the luminescence can be associated with defective recombination centers in the structure of forsterite [10,11,12] or enstatite [13]. In the works mentioned above, the shown spectra exhibited a broad band in the 350–550 nm range. It should be noted that the 2.75 eV (450 nm) emission is well known for α-quartz and thought to be an intrinsic property of SiO_4_ tetrahedrons, which are the main structural motifs in the olivines and pyroxenes [14].

**Figure 4:**
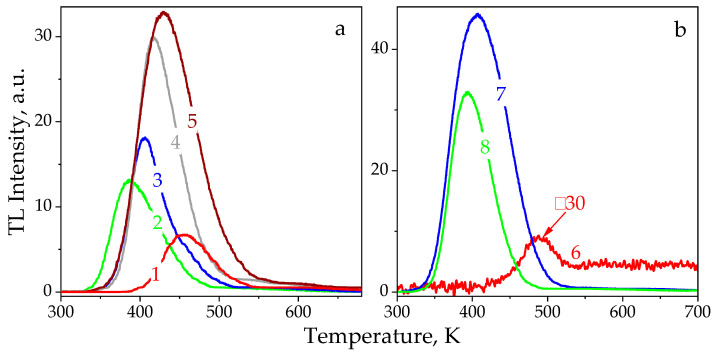
TL glow curves for meteorites irradiated with various doses. (**a**) The Chelyabinsk chondrite: 1—natural, 2—9.1 kGy, 3—18.2 kGy, 4—27.3 kGy, and 5—36.4 kGy. (**b**) The Tsarev chondrite: 6—natural, 7—9.1 kGy, and 8—9.1 kGy after 3 days of storage in the dark.

Figure 4 shows natural and induced TL curves for the studied samples. Regarding the Chelyabinsk LL5 chondrite, a maximum level of the natural TL was visible at 400–520 K. Apart from this, a high-temperature shoulder at 520–750 K was revealed. It is worth noting that the Dhajala meteorite showcases similar parameters for the main TL peak [5]. The emission demonstrates two ten-fold intensity changes, as the maximum temperature of the TL peak shifts from T_max_ = 427 to 487 K for different samples. This can be due to inhomogeneous mineral phases or mineral compositions, or due to differences in irradiation space doses [4]. The estimated values of the shape parameters for the TL curves measured for the chondrites under study are presented in Table 2. It can be noted that the high temperature shift of the induced TL peaks with increased doses was observed within the interval of T_max_ = (390–430) ± 10 K, while the halfwidths change was observed at ω_T_ = 61–83 K. The maximum temperatures of the natural TL peaks are noticeably higher for both meteorites.

The observed dependencies of the TL curve parameters on the irradiation dose cannot be described under the assumption made for independent charge-capturing centers, particularly in the frame of the “one trap—one recombination center” model [16]. The high values calculated for a geometric factor of μ_g_ = 0.58–0.64 indicate the processes with the kinetics order of b > 2 and are consistent with the estimates performed earlier in [17]. These facts evince the possible presence of a quasi-continuous system of capturing levels, which are active and interact in the investigated temperature range. Such a situation is quite typical for silicates (pyroxene, olivine, and others), in which various structural defects form luminescent complexes to be responsible for the processes analyzed.

The natural TL glow curve for the Tsarev L5 chondrite was found to contain a low intensity peak with a maximum temperature of T_max_ = 490 K and a halfwidth of w_T_ = 60 K (see Figure 4b and Table 2). In addition, storage time affects the induced TL maximum; it shifts to lower temperatures from 408 ± 5 to 394 ± 5 K. In this case, the halfwidth narrows from 86 ± 5 to 62 ± 5 K. The observed TL faded away by 45% after a 3-day holding period. For drawing a more reliable conclusion concerning the number of different capturing centers emptied within the RT–500 K temperature range, it is necessary to use additional TL techniques, such as dose or heating rate variation, step pre-heating, etc. [2,18].

In the case of measuring the natural TL, the equilibrium signal with information about the accumulated dose is assumed to be read from a trap, specifically the last emptying and deepest one. Accordingly, the natural glow curves obtained were analyzed in terms of the peak shape formalism for the general order kinetics [18]:

(1) $$E_{A}=\left[0.976+7.3\left(\mu_{g}-0.42\right)\right]\frac{kT_{\max}^{2}}{\delta },$$

Here, μ_g_ is the geometrical factor; δ is the high temperature halfwidth of the TL peak; k is the Boltzmann constant; and J·K^−1^ and E_A_ are the activation energies in eV. The values of E_A_ = 0.86 ± 0.10 and 1.08 ± 0.10 eV were calculated using the natural TL curves for the Chelyabinsk and Tsarev chondrites, respectively. The data obtained are in satisfactory agreement with E_A_ = 0.9–1.6 eV, which was taken from an analysis of the Dhajala meteorite thermoluminescence [5]. For the induced TL approximation, a superposition of several glow peaks, which characterized the presence of a quasi-continuous system of traps, should be used. We have no sufficient information to choose the number of the kinetic components.

## 4. Conclusions

The emission spectra, specifically the natural and induced TL glow curves in the 300–650 nm range, were measured for the Chelyabinsk and Tsarev stony meteorites using a luminescent spectrometer with a developed high-temperature appliance. All the TL spectra were approximated by a superposition of two Gaussians with maximum energies near 2.8 and 2.5 eV. The 2.8 eV band dominated in the TL emission and had an intensity four to six times higher than that of the 2.5 eV band. The conducted analysis of the obtained and independent data on the spectral parameters of PL and CL allows one to conclude that the observed features of the luminescence can be caused by defective recombination centers in the structure of forsterite in the meteorite composition.

The study of induced TL has shown that a high-temperature shift of ≈40 K is observed for the TL peak maximum as the dose increases within the 9.1–36.4 kGy range. We have revealed that a quasi-continuous traps distribution is active at 350–650 K. Based on the general order kinetics and using natural TL data for the Chelyabinsk and Tsarev meteorites, we also estimated the values of activation energies E_A_ = 0.86 ± 0.10 and 1.08 ± 0.10 eV. This work has demonstrated that thermoluminescence processes in the Chelyabinsk LL5 and Tsarev L5 chondrites are characterized by similar spectral and kinetic peculiarities.

## Figures and Tables

**Table 1:** Spectral parameters of luminescence for chondrites.

Chondrite	Method	T, K	E_max_, ±0.05 eV	ω_E_, ±0.05 eV	Reference
Chelyabinsk	TL	393	2.81 2.43	0.68 0.40	This work
	PL	RT	2.80 2.45	0.70 0.37	[6]
	CL	RT	2.68	0.75	[15]
Tsarev	TL	368	2.81 2.47	0.70 0.42	This work
		393	2.85 2.50	0.72 0.44	
		418	2.87 2.50	0.73 0.42	
		443	2.87 2.48	0.74 0.33	
	PL	RT	2.90 2.48	0.85 0.42	[9]

**Table 2:** TL parameters of chondrites after irradiation.

Chondrite	Dose, kGy	T_max_, K	ω_Τ_, K	μ_g_
Chelyabinsk	9.1	387	70	0.64
	18.2	402	66	0.62
	27.3	418	61	0.59
	36.4	429	83	0.58
	natural	456	72	0.56
Tsarev	9.1	408	86	0.53
	9.1 ^1^	394	62	0.58
	natural	490	60	0.50

^1^ after 3 days of storage in the dark.

## Data Availability

The data presented in this study are available on request from the corresponding author.
